# SoluProt: prediction of soluble protein expression in *Escherichia coli*

**DOI:** 10.1093/bioinformatics/btaa1102

**Published:** 2021-01-08

**Authors:** Jiri Hon, Martin Marusiak, Tomas Martinek, Antonin Kunka, Jaroslav Zendulka, David Bednar, Jiri Damborsky

**Affiliations:** Loschmidt Laboratories, Centre for Toxic Compounds in the Environment RECETOX and Department of Experimental Biology, Faculty of Science, Masaryk University, Brno 625 00, Czech Republic; International Clinical Research Center, St. Anne’s University Hospital Brno, Brno 656 91, Czech Republic; IT4Innovations Centre of Excellence, Faculty of Information Technology, Brno University of Technology, Brno 612 66, Czech Republic; IT4Innovations Centre of Excellence, Faculty of Information Technology, Brno University of Technology, Brno 612 66, Czech Republic; IT4Innovations Centre of Excellence, Faculty of Information Technology, Brno University of Technology, Brno 612 66, Czech Republic; Loschmidt Laboratories, Centre for Toxic Compounds in the Environment RECETOX and Department of Experimental Biology, Faculty of Science, Masaryk University, Brno 625 00, Czech Republic; International Clinical Research Center, St. Anne’s University Hospital Brno, Brno 656 91, Czech Republic; IT4Innovations Centre of Excellence, Faculty of Information Technology, Brno University of Technology, Brno 612 66, Czech Republic; Loschmidt Laboratories, Centre for Toxic Compounds in the Environment RECETOX and Department of Experimental Biology, Faculty of Science, Masaryk University, Brno 625 00, Czech Republic; International Clinical Research Center, St. Anne’s University Hospital Brno, Brno 656 91, Czech Republic; Loschmidt Laboratories, Centre for Toxic Compounds in the Environment RECETOX and Department of Experimental Biology, Faculty of Science, Masaryk University, Brno 625 00, Czech Republic; International Clinical Research Center, St. Anne’s University Hospital Brno, Brno 656 91, Czech Republic

## Abstract

**Motivation:**

Poor protein solubility hinders the production of many therapeutic and industrially useful proteins. Experimental efforts to increase solubility are plagued by low success rates and often reduce biological activity. Computational prediction of protein expressibility and solubility in *Escherichia coli* using only sequence information could reduce the cost of experimental studies by enabling prioritization of highly soluble proteins.

**Results:**

A new tool for sequence-based prediction of soluble protein expression in *E.coli*, SoluProt, was created using the gradient boosting machine technique with the TargetTrack database as a training set. When evaluated against a balanced independent test set derived from the NESG database, SoluProt’s accuracy of 58.5% and AUC of 0.62 exceeded those of a suite of alternative solubility prediction tools. There is also evidence that it could significantly increase the success rate of experimental protein studies. SoluProt is freely available as a standalone program and a user-friendly webserver at https://loschmidt.chemi.muni.cz/soluprot/.

**Availability and implementation:**

https://loschmidt.chemi.muni.cz/soluprot/.

**Supplementary information:**

Supplementary data are available at *Bioinformatics* online.

## 1 Introduction

Low protein solubility causes severe problems in protein science and industry; insufficient protein solubility is probably the most common cause of failure of protein production pipelines. The importance of solubility is underlined by the findings of the large-scale Protein Structure Initiative (PSI) project (Berman *et al.*, 2017), which sought to produce thousands of protein sequences from different organisms, crystallize them and resolve their tertiary structure. Unfortunately, in most cases it proved impossible to produce the target proteins in soluble form. The inherent low solubility of natural enzymes also limits the success of emerging high-throughput pipelines that explore protein databases to identify novel enzymes with diverse functions (Hon *et al.*, 2020; Vanacek *et al.*, 2018). Given the rapid growth of protein sequence databases driven by the capabilities of next-generation sequencing technologies, there is an urgent need to focus only on potentially soluble targets to avoid wasting resources on hard-to-produce orthologs. Solubility is thus a key attribute when choosing protein targets for experimental characterization (Vanacek *et al.*, 2018). Strictly speaking, solubility is a thermodynamic parameter defined as the protein’s concentration in a saturated solution in equilibrium with a solid phase under specific conditions. However, it is challenging to quantitatively measure the solubility of large sets of proteins (Kramer *et al.*, 2012), so there is little quantitative experimental data on protein solubility. Moreover, this definition of solubility is too narrow to encompass many of the practical problems that may occur during protein production with common expression systems. Therefore, inspired by existing tools (Supplementary Table S1) (Agostini *et al.*, 2014; Khurana *et al.*, 2018; Raimondi *et al.*, 2020; Smialowski *et al.*, 2012), available data (Berman *et al.*, 2017) and laboratory practice, we use a slightly extended definition of protein solubility in this work. Specifically, by solubility, we mean the probability of soluble protein (over)expression in *Escherichia coli* cells. The difference from the classical thermodynamic solubility is in the perception of the insoluble class. We assume that insoluble proteins were either not expressed or were expressed in the insoluble form.

Solubility depends on many extrinsic and intrinsic factors. Extrinsic factors are dictated by the choice of expression system and the experimental conditions used in protein production. Expression systems may be either *in vivo* or *in vitro* (Carlson *et al.*, 2012; Rosano and Ceccarelli, 2014). *In vivo* protein expression is induced inside living cells of a host organism, whereas *in vitro* expression relies on the use of cell-free translational systems. Solubility can be increased by adjusting extrinsic solubility factors, especially by using different mutated host strains, codon optimization, coexpression of chaperones and foldases, lowering cultivation temperatures and adding suitable fusion partners (Costa *et al.*, 2014). However, tuning the expression system or experimental conditions is not always sufficient to confer solubility, and is not feasible in high-throughput protein production pipelines. If extrinsic factors cannot be varied, protein solubility will depend only on the intrinsic properties of the protein sequence. Unfortunately, the relationship between a protein’s sequence and its solubility is poorly understood, mainly due to a lack of reproducible quantitative solubility measurements (Kramer *et al.*, 2012). Recent protein engineering studies suggest that charged amino acids on the protein surface are key intrinsic determinants of solubility (Carballo-Amador *et al.*, 2019; Chan *et al.*, 2013; Sankar *et al.*, 2018). However, this knowledge cannot be directly used for solubility prediction due to a lack of structural data. Despite the continuous growth of structural databases (Burley *et al.*, 2019), the structures of proteins of interest are generally unknown, and the limited availability of template structures prevents their accurate computational prediction.

The simultaneous effects of extrinsic and intrinsic factors make solubility prediction challenging. For example, the prediction of solubility from sequence data using machine learning is hindered by the high level of noise in typical training datasets due to the influence of diverse extrinsic variables. Because the molecular mechanisms governing protein solubility are poorly understood, recent solubility prediction tools rely heavily on statistical analysis and machine learning, using previously reported experimental data to train and validate model parameters. One of the most widely used data sources is the TargetTrack database (Berman *et al.*, 2017), formerly known as PepcDB or TargetDB, which integrates information from the Protein Structure Initiative projects. This database contains data from over 900 000 protein crystallization trials involving almost 300 000 unique protein sequences, which are referred to as targets. The database does not contain solubility data per se, but target proteins can be considered soluble if they were successfully purified in the experimental trials. A major limitation of this database is the low quality of its annotations. For example, reasons for failure are generally not provided for unsuccessful crystallization attempts. Therefore, it is impossible to distinguish failures due to insolubility from failures due to other problems later in the experimental pipeline. Second, the experimental protocols used for protein production and crystallization are described in free text with no internal structure, making it hard to automatically extract information about experimental conditions and expression systems for a given target. Filtering is therefore needed to reduce noise before using the TargetTrack data for model training. However, the application of stringent filtering rules to the target annotations can dramatically reduce the number of usable records.

eSOL is another well-known and commonly used solubility database (Niwa *et al.*, 2009, 2012) that contains experimentally measured solubilities for over 3 000 *E.coli* proteins produced in the PURE (Shimizu *et al.*, 2001) cell-free expression system. eSOL is an impressive collection of highly homogenous data but has its own limitations. First, it only contains data on proteins originating from *E.coli.* Second, it has relatively little negative data; adding the three main cytosolic *E.coli* chaperones (TF, DnaKJE and GroEL/GroES) to the PURE expression system reduced the number of insoluble proteins from 788 to 24 (Niwa *et al.*, 2012). eSOL is a valuable source of exact solubility data that were generated using a robust pipeline and provide a good quantitative measure of thermodynamic solubility. However, these data cannot be used to assess solubility according to our expanded definition, which also encompasses expressibility.

The relationship between protein sequence and solubility has been studied for over 30 years, leading to the development of several predictive models and software tools. There are 11 such models or tools that use definitions of solubility like that described above and take protein sequences as their sole input. These are the revised Wilkinson-Harrison model (rWH) (Davis *et al.*, 1999; Wilkinson and Harrison, 1991), SOLpro (Magnan *et al.*, 2009), RPSP (Diaz *et al.*, 2010), PROSO II (Smialowski *et al.*, 2012), ccSOL omics (Agostini *et al.*, 2012, 2014), ESPRESSO (Hirose and Noguchi, 2013), CamSol (Sormanni *et al.*, 2015), Protein-Sol (Hebditch *et al.*, 2017), DeepSol (Khurana *et al.*, 2018), SKADE (Raimondi *et al.*, 2020) and the Solubility-weighted index (SWI) (Bhandari *et al.*, 2020). However, the accuracy of these tools is limited, and there is clear room for improvement. Additionally, these tools exhibit poor generality when used to make predictions based on previously unseen data. A comprehensive review of advances in solubility prediction, including predictors that use protein structures as inputs, was published recently (Musil *et al.*, 2019). Here, we present a novel machine learning based tool, SoluProt, for predicting soluble expression from protein sequence data. SoluProt benefits from thorough dataset pre-processing and predicts soluble expression more accurately than previously reported methods.

## 2 SoluProt training and test set

We used the TargetTrack database to build the *SoluProt training set*. Since this database does not directly provide solubility information, we inferred solubility computationally, using an approach similar to those adopted previously (Magnan *et al.*, 2009; Smialowski *et al.*, 2012). A protein was considered *soluble* if it was recorded as having reached a soluble experimental state or any subsequent state requiring soluble expression (Supplementary Table S2). If failed expression or purification was mentioned in the experiment record's stop status, the protein was labeled *insoluble*. In contrast to a previous approach (Smialowski *et al.*, 2012), we required an explicit stop status relating to insolubility to reduce the frequency of incorrect classification of insoluble sequences. To improve the quality of the training set, we also performed several additional steps to clean the data.

Most importantly, we performed keyword matching combined with manual checking of TargetTrack annotations to extract only proteins expressed in the most common host organism, *E.coli*. This was necessary because a protein soluble in one organism might be insoluble in another. By focusing solely on the most common expression system, we reduced the noise in the training data. We also used specific keywords to search the unstructured descriptions of experimental protocols provided in the TargetTrack database (Supplementary Table S3). Generic search phrases like ‘*E.coli*’ or ‘*Escherichia coli*’ were used to identify potential *E.coli* related protocols. These protocols were then manually checked and confirmed (Supplementary Table S4). A full list of 248 TargetTrack protocols signifying expression in *E.coli* is available at the SoluProt website.

We next identified transmembrane proteins in the dataset based on direct annotations from the TargetTrack database and predictions generated using TOPCONS (Tsirigos *et al.*, 2015) with default settings. The transmembrane proteins were then removed, along with sequences shorter than 20 amino acids, and sequences with undefined residues. We also removed sequences that had been classified as insoluble but for which a protein structure was available in the Protein Data Bank (PDB) (Berman, 2000). To this end, we compiled an *E.coli* PDB subset containing sequences of proteins whose structures had been solved by NMR or X-ray crystallography and which had been expressed in *E.coli* according to the PDB annotations (64 416 sequences, downloaded April 4, 2018). Because both NMR and X-ray crystallography require soluble proteins, any protein in this PDB subset can be considered soluble in *E.coli*. This step reflects advances in molecular biology: methodological developments have made it possible to produce and crystallize some proteins that were previously considered insoluble.

Finally, we reduced the sequence redundancy in the training set by clustering to 25% identity using MMseqs2 (Steinegger and Söding, 2017) and retaining only representative sequences from each cluster. This was done separately for positive and negative samples to avoid simplifying the prediction problem. We balanced the number of soluble and insoluble samples such that both classes were equally represented. Additionally, we balanced the sequence length distribution so that length alone would not play a dominant role in the predictions. Sequence length correlates with protein solubility—larger proteins are usually less soluble. However, we wanted to suppress its influence in the model because we anticipate that SoluProt would mainly be used to prioritize proteins of similar lengths, usually from a single protein family. A typical expected use case is that of the EnzymeMiner web server (Hon *et al.*, 2020) for automated mining of soluble enzymes. A prediction model relying heavily on sequence length would not perform well in this use case.

The *SoluProt test set* was built from a dataset generated by the North East Structural Consortium (NESG), which represents 9644 proteins expressed in *E.coli* using a unified production pipeline (Price *et al.*, 2011). The dataset contains two integer scores ranging from 0 to 5 for each target, indicating the protein’s level of expression and the soluble fraction recovery. The reproducibility of the experimental results in the dataset was validated by performing repeat measurements for selected targets. The NESG dataset targets are included in the TargetTrack database because the NESG participated in the PSI project. However, the expression and solubility levels from the NESG dataset were not included in the TargetTrack database; instead, they were provided to us directly by the authors of the original study (W. Nicholson Price II, personal communication). The high consistency and quality of the NESG dataset make it suitable for benchmarking purposes. We processed the NESG dataset using the same procedure as the training set, although the computational solubility derivation and expression system filtration steps were omitted because they were pointless in this case. Instead, we transformed the solubility levels into binary classes: all proteins with a solubility level of 1 or above were considered soluble and all others insoluble.

Finally, we ensured that no pair consisting of a sequence from the test set and a sequence from the training set had a global sequence identity above 25% as calculated using the USEARCH software (Edgar, 2010). This made the test set more independent because it ensured that predictions were not validated against data similar to those used during training. In total, 11 436 protein sequences remained in the *SoluProt training set* and 3 100 in the independent *SoluProt test set*. Both datasets had equal numbers of soluble and insoluble samples with balanced sequence length distributions (Supplementary Fig. S1). The datasets are available at the SoluProt website. The dataset construction steps are summarized in Supplementary Table S5.

## 3 Prediction model

The SoluProt predictor is implemented in Python using scikit-learn (Pedregosa *et al.*, 2011), Biopython (Cock *et al.*, 2009) and pandas (McKinney, 2010) libraries. We used a gradient boosting machine (GBM) (Friedman, 2001) to generate the predictive model. Prediction features were selected from a set of 251 sequence characteristics that were divided into eight groups: (i) single amino acid content (20 features), (ii) amino acid dimer content (210 features), (iii), sequence physicochemical features (12 features, Supplementary Table S6), (iv) average flexibility as computed by DynaMine (Cilia *et al.*, 2014) (1 feature), (v) secondary structure content as predicted by FESS (Piovesan *et al.*, 2017) (3 features), (vi) average disorder as predicted by ESPRITZ (Walsh *et al.*, 2012) (1 feature), (vii) content of amino acids in transmembrane helices as predicted by TMHMM (Krogh *et al.*, 2001) (3 features) and (viii) maximum identity to the *E.coli* PDB subset as calculated using USEARCH (1 feature). All sequences equal to any sequence from the test set were excluded from the *E.coli* PDB subset for the calculation of maximum identity. The objective was to eliminate even the indirect presence of test set sequences from model training. We standardized all features by subtracting the mean and scaling to unit variance. The means and variances were calculated using the training set.

We removed correlated features in two steps. First, we fitted a GBM with default parameters using the full training set and all features. Second, we calculated Pearson’s correlation coefficient for each pair of features. If the correlation between any two features exceeded 0.75, we removed the feature with the lesser importance in the fitted GBM model. We also removed irrelevant features using LASSO (Tibshirani, 1996). LASSO's alpha parameter was optimized to maximize the mean AUC of the GBM model with default parameters over 5-fold cross-validation. The alpha parameter was varied between 0.08 to 0 with a step size of 6.25×10^−4^; its optimal value was 0.005. In total, 96 features were selected for inclusion in the predictive model (Supplementary Table S7). The DynaMine, FESS and ESPRITZ features were not included in the final feature set.

We next optimized the hyperparameters of the GBM model, using an iterative 7-stage strategy to maximize the mean AUC over 5-fold cross-validation using the training set (Supplementary Table S8). In each stage, one or two parameters were optimized using grid search; other parameters were left either at their final values from the previous stages or at the default value if the parameter had not yet been optimized. The best GBM model achieved mean AUC values of 0.85 ± 0.003 for the training part and 0.72 ± 0.02 for the validation part. Overall, the feature selection and hyperparameter optimization had little effect on the mean AUC: without these measures, the mean AUC values for the training and validation sets were 0.83 ± 0.003 and 0.72 ± 0.02, respectively. The main benefit of the feature selection and parameter tuning steps was that they reduced the number of features and thus made the feature calculation step roughly two times faster.

Finally, we used the best GBM hyperparameters to train the final SoluProt model using the full training set. The resulting model had an AUC of 0.84 and an accuracy of 76% for the full training set. The five most important features according to the GBM are: (i) maximum identity to the *E.coli* PDB subset (14.5%), (ii) isoelectric point (6.2%), (iii) predicted number of amino acids in transmembrane helices in the first sixty amino acids of the protein (4.2%), (iv) lysine content (4.0%) and (v) glutamine content (3.5%) (Supplementary Table S7).

## 4 Performance evaluation and comparison

**Fig. 1.**
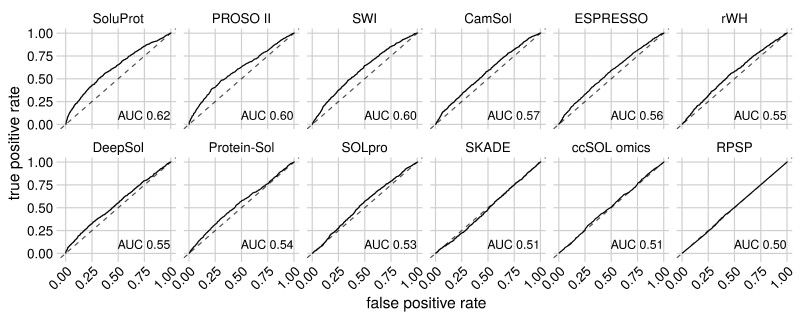
Receiver operating curves (ROC) calculated for the balanced SoluProt test set of 3100 sequences. The predictors are ordered by the area under the receiver operating curve (AUC)

We used the SoluProt test set to evaluate and compare SoluProt to 11 previously published tools. The evaluation relied on both threshold-independent (area under the ROC curve) and threshold-dependent metrics (accuracy, Matthew’s correlation coefficient and confusion matrices). For the threshold-dependent metrics, we applied a threshold of 0.5 or the thresholds recommended by the authors of the corresponding method (Table 1). SoluProt achieved the highest accuracy (58.5%) and the greatest AUC (0.62) of the tested tools when evaluated against the SoluProt test set (Table 1 and Fig. 1),followed by PROSO II and SWI.

**Table 1.** Performance of various solubility predictors using the balanced SoluProt test set of 3100 sequences

Method	AUC	T	ACC	MCC	TP	TN	FP	FN
SoluProt	0.62	0.50	58.5%	0.17	939	873	677	611
PROSO II	0.60	0.60	58.0%	0.17	630	1167	383	920
SWI	0.60	0.50	55.9%	0.13	1206	527	1023	344
CamSol	0.57	1.00	54.1%	0.08	676	1001	549	874
ESPRESSO	0.56	0.50	53.8%	0.08	1003	664	886	547
rWH	0.55	0.50	54.0%	0.08	670	1005	545	880
DeepSol	0.55	0.50	52.9%	0.09	230	1409	141	1320
Protein-Sol	0.54	0.45	51.6%	0.03	1056	544	1006	494
SOLpro	0.53	0.50	52.0%	0.04	654	959	591	896
SKADE	0.51	0.50	49.2%	–0.03	159	1366	184	1391
ccSOL omics	0.51	0.50	50.8%	0.02	884	690	860	666
RPSP	0.50	0.50	49.8%	0.00	501	1044	506	1049

*Note*: The different definitions of solubility and target expression system (Supplementary Table S1) should be considered when comparing the performance of individual tools.

AUC—area under the ROC curve, T—threshold for the soluble class, ACC—accuracy, MCC—Matthew’s correlation coefficient, TP—true positives, TN—true negatives, FP—false positives, FN—false negatives.

While the SoluProt test set is independent of the SoluProt training set, other tools' training sets might overlap with our test set. Therefore, we compared the SoluProt test set to the training sets of DeepSol, SKADE, SWI and SOLpro to quantify their overlaps (Table 2). DeepSol and SKADE have a common training set, which showed the largest overlap (74.0%), followed by the SWI training set (26.5%) and the SOLpro training set (15.5%). SWI benefits from the overlap; it was the third-best tool in our comparison. DeepSol and SKADE ranked 7th and 12th by accuracy with respect to the SoluProt test set despite having the greatest proportion of test sequences in their training set. This comparatively poor performance can be partly explained by differences in solubility annotations between the DeepSol training set and the SoluProt test set (Table 2): 360 (11.6% of the total) sequences annotated as insoluble in the DeepSol training set were annotated as soluble in the SoluProt test set. The total number of disagreements (the sum of false positives and false negatives) ranged from 336 to 551, depending on the binarization threshold applied to the SoluProt test set (Supplementary Table S9). No training set was published for PROSO II; only an initial set of soluble and insoluble sequences without pre-processing is available. However, the initial set exhibits 95.2% overlap with the SoluProt test set. Therefore, we expect the overlap of the PROSO II training set to also be very high, like the DeepSol training set. Unfortunately, the training sets of other previously developed tools have not been published, preventing a more comprehensive comparison.

**Table 2.** Overlaps between the SoluProt test set and available training sets

Dataset	Size	Test set overlap	TP	TN	FP	FN
*PROSO II initial*	129643	2952 (95.2%)	951	1437	50	514
DeepSol/SKADE	69420	2294 (74.0%)	737	1130	67	360
SWI	12216	820 (26.5%)	537	210	53	20
SOLpro	17408	480 (15.5%)	178	120	39	143

*Note*: Two sequences were considered identical if their global sequence identity reported by USEARCH was 100%. Differences in solubility annotations for identical sequences were quantified using confusion matrix terms (TP, TN, FP and FN). The solubility annotations of the SoluProt test set are assumed to reflect the true solubilities of the proteins.

TP—true positives, TN—true negatives, FP—false positives, FN—false negatives. ^a^ DeepSol and SKADE share the same training set.

**Fig. 2.**
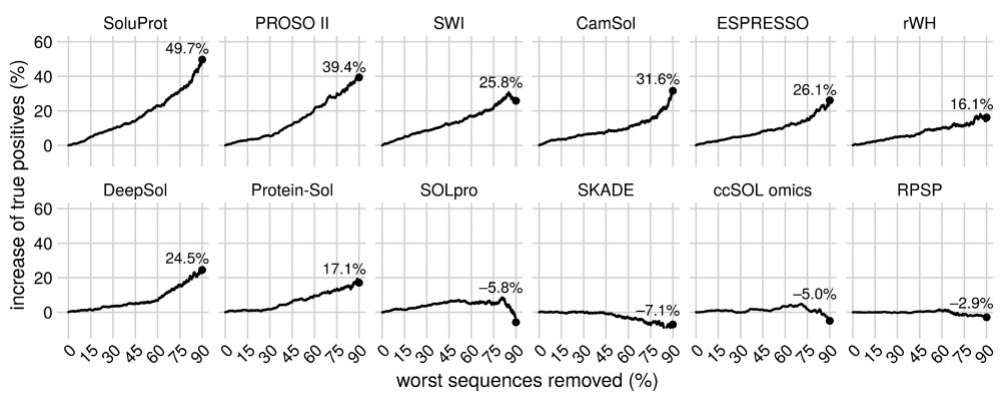
Increases in the number of true positives resulting from sequence prioritization using the tested solubility prediction tools. The SoluProt test set sequences were ordered by predicted solubility based on each predictor’s output, and a variable percentage of the sequences with the worst predicted solubility was then removed. The increase in the number of true positives was then calculated relative to a baseline random selection. For example, upon randomly removing 90% of the test set sequences (2790 samples), we would expect half of the remaining 310 sequences to be true positives

The absolute accuracy of the available solubility prediction tools is low (below 60%), so there is clearly room for improvement. Nevertheless, SoluProt and other tools can be useful for protein sequence prioritization (Fig. 2), i.e. for selecting a small number of sequences for in-depth experimental characterization from a large database of several hundreds or thousands of sequences. Specifically, predicted solubility values can be used to select a limited number of high-scoring protein sequences. For example, if we use SoluProt predictions to order the SoluProt test set and remove all sequences bar the 10% with the highest scores, we get 232 true positives, i.e. 49.7% more true positives than would be expected with blind selection (155 true positives). This shows that despite their limited accuracy, current solubility predictors are valuable for protein sequence prioritization and can increase the success rate of experimental protein studies.

## 5 Conclusions

We have developed a novel method and software tool, SoluProt, for sequence-based prediction of soluble protein expression in *E.coli*. The tool simultaneously predicts the solubility and expressibility of the proteins under consideration. SoluProt achieved a higher accuracy (58.5%) and AUC (0.62) than a suite of alternative solubility prediction tools when evaluated using the balanced independent SoluProt test set of 3100 sequences. PROSO II, SWI and CamSol were the next best tools, achieving accuracies of 58.0%, 55.9% and 54.1%, respectively. SoluProt also performed well in protein prioritization. The main strengths of SoluProt are that it was trained using a dataset generated by thorough pre-processing of the noisy TargetTrack data, and was validated using a high-quality independent test set.

Surprisingly, the recently reported DeepSol (Khurana *et al.*, 2018) and SKADE (Raimondi *et al.*, 2020) tools, which are based on deep learning methods, performed worse than the simpler and mostly older methods PROSO II (Smialowski *et al.*, 2012), SWI (Bhandari *et al.*, 2020) and CamSol (Sormanni *et al.*, 2015) in our comparison. This may be partly due to the overlap of their training set with our test set and disagreements between these sets with respect to the solubility of certain sequences.

The SoluProt predictor is available via a user-friendly web server or as a standalone software package at https://loschmidt.chemi.muni.cz/soluprot/. The SoluProt web server has already predicted the solubility of over 4700 unique protein sequences in ten months since its launch in February 2020. It has also been integrated into the web server EnzymeMiner (Hon *et al.*, 2020) for automated mining of novel soluble enzymes from protein databases (https://loschmidt.chemi.muni.cz/enzymeminer/).

## Funding

This work was supported by Czech Ministry of Education [857560, 02.1.01/0.0/0.0/18_046/0015975, CZ.02.1.01/0.0/0.0/16_026/0008451, LQ1602]; Czech Grant Agency (20-15915Y); European Commission [857560, 720776, 814418]; and AI Methods for Cybersecurity and Control Systems project of the Brno University of Technology [FIT-S-20-6293]. Computational resources were supplied by the project ’e-Infrastruktura CZ’ [e-INFRA LM2018140] and by the ELIXIR-CZ [LM2018131]. Funding for open access charge: Czech Ministry of Education.

*Conflict of Interest*: none declared.

## Supplementary Material

btaa1102_Supplementary_Data
