# Supplementary material for: SoluProt: prediction of soluble protein expression in Escherichia coli
Source: Bioinformatics. 2021 Jan 8;37(1):23–8. doi: 10.1093/bioinformatics/btaa1102 (PMC8034534; doi:10.1093/bioinformatics/btaa1102)
Supplement: btaa1102_Supplementary_Data [file btaa1102_supplementary_data.zip › Hon_Bioinformatics_SI.docx]

#

#

# Supplementary Information

# SoluProt: Prediction of Soluble Protein Expression in *Escherichia coli*

Jiri Hon^1,2,3^, Martin Marusiak^3^, Tomas Martinek^3^, Antonin Kunka^1,2^, Jaroslav Zendulka^3^, David Bednar^1,2^, Jiri Damborsky^1,2^

^1^Loschmidt Laboratories, Centre for Toxic Compounds in the Environment RECETOX and Department of Experimental Biology, Faculty of Science, Masaryk University, 625 00 Brno, Czech Republic; ^2^International Clinical Research Center, St. Anne’s University Hospital Brno, 656 91 Brno, Czech Republic; ^3^IT4Innovations Centre of Excellence, Faculty of Information Technology, Brno University of Technology, 612 66 Brno, Czech Republic

Table S1. The summary of solubility concepts used in existing tools. The key difference lies in the perception of the insoluble class. The tools predicting “soluble expression” assume that insoluble proteins were either not expressed or were expressed in the insoluble form. The tools predicting classical “solubility” assume that insoluble proteins were successfully expressed before the solubility was determined.

| **Method** | **Predicted property** | **Expression system** | **Comment** |
| --- | --- | --- | --- |
| SoluProt | soluble expression | *E. coli* | Based on curated TargetTrack data. |
| PROSO II | soluble expression | mixed | Based on PepcDB data (predecessor of TargetTrack). |
| SWI | solubility | *E. coli* | Based on PSI:Biology data. All proteins in the dataset were successfully expressed. |
| CamSol | solubility | mixed | Based on literature data. |
| ESPRESSO | solubility | *E. coli* | Based on Hirose dataset (Hirose *et al.*, 2011). |
| rWH | solubility | *E. coli* | Based on literature data. |
| DeepSol | soluble expression | mixed | Based on TargetTrack data. |
| Protein-Sol | solubility | cell-free | Based on eSOL data. |
| SOLpro | solubility | mixed | Based on PDB, Swiss-Prot and TargetTrack data. Proteins marked as insoluble were required to reach at least “cloned” or “expressed” states. |
| SKADE | soluble expression | mixed | Based on TargetTrack data. |
| ccSOL omics | soluble expression | mixed | Based on TargetTrack data. |
| RPSP | solubility | *E. coli* | Based on literature data. |

Table S2. TargetTrack experiment states signifying soluble expression. The list was compiled by the authors of PROSO II (Smialowski *et al.*, 2012).

| **Experiment states** |
| --- |
| soluble, purified, crystallized, hsqc, structure, in pdb, native diffraction-data, NMR assigned, phasing diffraction-data, diffraction, in bmrb, nmr structure, crystal structure, diffraction-quality crystals |

Table S3. Specific keywords signifying expression in *E. coli*.

| **Specific keywords** |
| --- |
| BL21, DE3, rosetta, xl10, DH10B, CodonPlus, RIPL, RIL, DB3.1, DB3, arctic, origami |

Table S4. Protocols identified by generic phrases and manually checked to signify expression in *E.coli*.

| **Protocol id** |
| --- |
| NYSGXRC-SGX_MOLBIO_TOPO_TRANSFORM  JCSG-E_Ecoli_GNF_1  CSGID-NU_SelMet_expression  CSGID-NU_native_expression  MPP-LP.4341  MCSG-NU_default_expression  NYSGXRC-SGX_FERM_ECOLI_LB  MPP-LP.4813  SSGCID-33  NYSGXRC-SGX_FERM_ECOLI_M9  CSGID-NU_default_expression  SSGCID-2  SSGCID-31  SSGCID-1  CESG-MAXWELL 16 EXPRESSION TESTING (R D) v.1.0.0  MPP-LP.4814  SSGCID-128  EFI-SeMET expression in HY Media-PSI2  SGX-SGX_FERM_ECOLI_LB_CFTR  SGX-SGX_MOLBIO_EXPR_SOL_CFTR |

Table S5. The number of sequences retained in each dataset construction step. The higher number of soluble sequences in comparison to insoluble sequences in the training set can be explained by the lack of stop status annotation in the TargetTrack database. Therefore, it is generally harder to reliably extract insoluble sequences from the TargetTrack database.

| **Construction step** | **Training set** | **Soluble** | **Insoluble** | **Test set** | **Soluble** | **Insoluble** |
| --- | --- | --- | --- | --- | --- | --- |
| Input | 335,771^T^ | - | - | 9,703^R^ | - | - |
| Pre-processing and solubility assignment | 114,648^R^ | - | - | - | - | - |
| Expression system detection | 82,362^R^ | - | - | - | - | - |
| Redundancy removal | 54,969 | 40,905 | 14,064 | 9,423 | 5,718 | 3,705 |
| Removal of short sequences and sequences with unknown residues | 54,962 | 40,904 | 14,058 | 9,420 | 5,715 | 3,705 |
| Removal of transmembrane proteins | 51,380 | 38,633 | 12,747 | 8,769 | 5,421 | 3,348 |
| Removal of insoluble sequences with available PDB structure | 51,360 | 38,633 | 12,727 | 8,754 | 5,421 | 3,333 |
| Overlap removal^a^ | - | - | - | 6,398 | 3.928 | 2,470 |
| Clustering to 25% identity | 22,169 | 16,422 | 5,747 | 3,545 | 1,990 | 1,555 |
| Class and length balancing | **11,436** | 5,718 | 5,718 | **3,100** | 1,550 | 1,550 |

^T^The number of targets in the TargetTrack database. ^R^The number of extracted sequence records – possibly more than one record for a sequence. Without any superscript – the number of unique protein sequences.

^a^Test set sequences sharing >25% sequence identity to any training set sequence were removed. The input for this step was the final training set of 11,436 sequences to minimize the reduction of the test set.


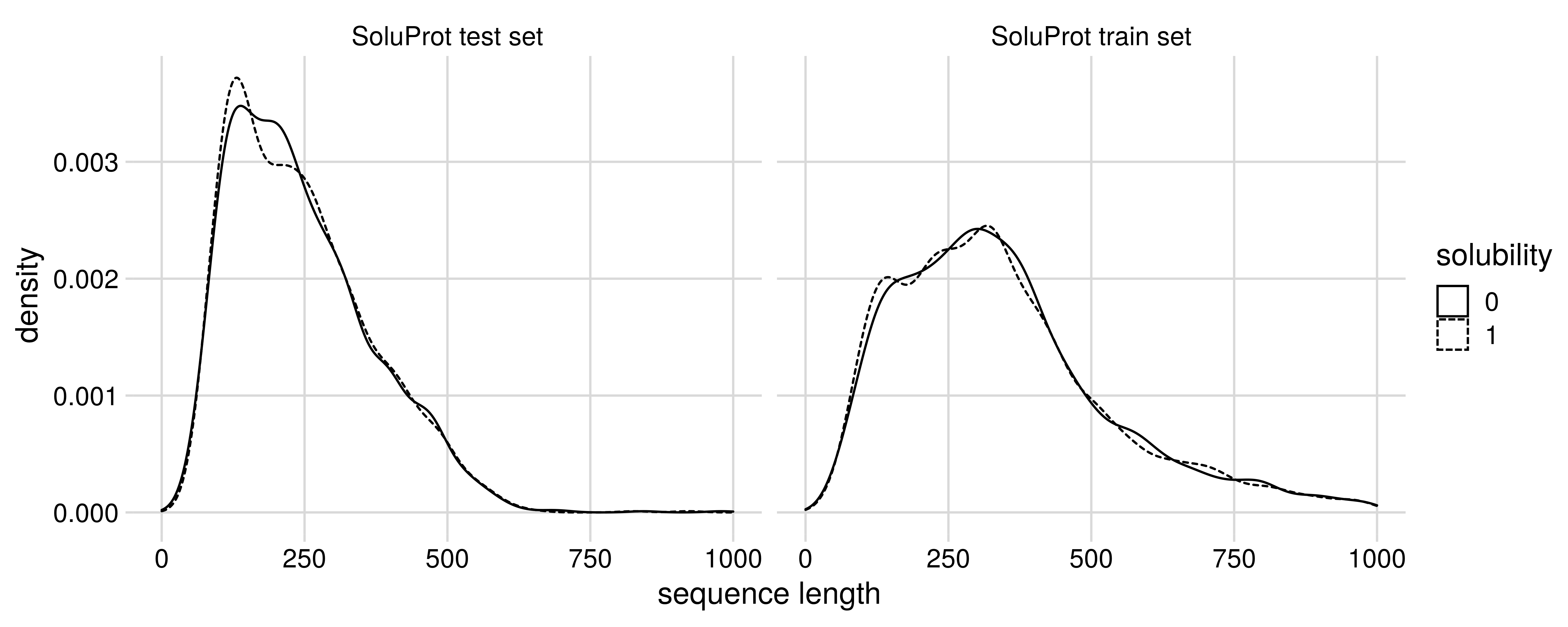


Figure S1. Sequence length distribution of soluble and insoluble proteins in the SoluProt datasets. The x-axis is limited to the range of 0–1,000 amino acids to improve readability. The longest sequences in the test and training sets have 979 and 2,825 amino acids, respectively.

Table S6. Sequence physicochemical features. Most of the features were extracted using the Biopython package (Cock *et al.*, 2009).

| **Feature** | **Description** |
| --- | --- |
| physico_chemical_fracnumcharge | Fraction of charged amino acids (R, K, D, E). |
| physico_chemical_kr_ratio | Ratio of K and R content. |
| physico_chemical_aa_helix | Fraction of helix amino acids (V, I, Y, F, W, L). |
| physico_chemical_aa_sheet | Fraction of sheet amino acids (E, M, A, L). |
| physico_chemical_aa_turn | Fraction of turn amino acids (N, P, G, S). |
| physico_chemical_molecular_weight | Molecular weight. |
| physico_chemical_avg_molecular_weight | Molecular weight normalized by the sequence length. |
| physico_chemical_aromaticity | Fraction of aromatic amino acids (Y, W, F) |
| physico_chemical_flexibility | Flexibility according to (Vihinen *et al.*, 1994) |
| physico_chemical_gravy | Grand average of hydropathy according to (Kyte and Doolittle, 1982) |
| physico_chemical_isoelectric_point | Isoelectric point using methods of Bjellqvist (Bjellqvist *et al.*, 1993, 1994) |
| physico_chemical_instability_index | Instability index according to (Guruprasad *et al.*, 1990) |

Table S7. Sequence features and their importance in the final SoluProt model.

| **#** | **Feature** | **Importance** |  | **#** | **Feature** | **Importance** |
| --- | --- | --- | --- | --- | --- | --- |
| 1 | ecoli_usearch_identity_identity | 14.54% |  | 26 | dimers_comb_EM | 0.96% |
| 2 | physico_chemical_isoelectric_point | 6.23% |  | 27 | monomers_F | 0.94% |
| 3 | tmhmm_first_60 | 4.18% |  | 28 | dimers_comb_EN | 0.92% |
| 4 | monomers_K | 3.95% |  | 29 | dimers_comb_AV | 0.89% |
| 5 | monomers_Q | 3.48% |  | 30 | dimers_comb_DL | 0.89% |
| 6 | physico_chemical_aa_helix | 1.91% |  | 31 | dimers_comb_IS | 0.87% |
| 7 | monomers_E | 1.84% |  | 32 | dimers_comb_EE | 0.86% |
| 8 | physico_chemical_molecular_weight | 1.77% |  | 33 | dimers_comb_CG | 0.85% |
| 9 | monomers_M | 1.70% |  | 34 | dimers_comb_PQ | 0.85% |
| 10 | dimers_comb_DK | 1.57% |  | 35 | dimers_comb_LQ | 0.83% |
| 11 | dimers_comb_RR | 1.55% |  | 36 | dimers_comb_EH | 0.82% |
| 12 | dimers_comb_EK | 1.49% |  | 37 | dimers_comb_AQ | 0.82% |
| 13 | monomers_Y | 1.39% |  | 38 | monomers_H | 0.82% |
| 14 | dimers_comb_AA | 1.35% |  | 39 | dimers_comb_CI | 0.79% |
| 15 | monomers_C | 1.28% |  | 40 | dimers_comb_EL | 0.79% |
| 16 | dimers_comb_GK | 1.13% |  | 41 | dimers_comb_HT | 0.78% |
| 17 | dimers_comb_DT | 1.09% |  | 42 | dimers_comb_EI | 0.77% |
| 18 | dimers_comb_LN | 1.09% |  | 43 | dimers_comb_QV | 0.76% |
| 19 | dimers_comb_FT | 1.08% |  | 44 | dimers_comb_DE | 0.75% |
| 20 | dimers_comb_AI | 1.05% |  | 45 | dimers_comb_DM | 0.74% |
| 21 | dimers_comb_DI | 1.02% |  | 46 | dimers_comb_MV | 0.74% |
| 22 | dimers_comb_AG | 1.01% |  | 47 | dimers_comb_GL | 0.74% |
| 23 | dimers_comb_LT | 1.00% |  | 48 | monomers_W | 0.73% |
| 24 | dimers_comb_MN | 0.98% |  | 49 | dimers_comb_TY | 0.72% |
| 25 | dimers_comb_AN | 0.98% |  | 50 | physico_chemical_fracnumcharge | 0.72% |

| **#** | **Feature** | **Importance** |  | **#** | **Feature** | **Importance** |
| --- | --- | --- | --- | --- | --- | --- |
| 51 | dimers_comb_EV | 0.70% |  | 74 | dimers_comb_CS | 0.48% |
| 52 | dimers_comb_SV | 0.65% |  | 75 | dimers_comb_CP | 0.47% |
| 53 | dimers_comb_RW | 0.65% |  | 76 | dimers_comb_AK | 0.47% |
| 54 | dimers_comb_QT | 0.64% |  | 77 | dimers_comb_IY | 0.46% |
| 55 | dimers_comb_KQ | 0.61% |  | 78 | dimers_comb_PW | 0.45% |
| 56 | dimers_comb_GV | 0.61% |  | 79 | dimers_comb_VY | 0.45% |
| 57 | dimers_comb_KV | 0.60% |  | 80 | dimers_comb_NY | 0.43% |
| 58 | dimers_comb_HL | 0.59% |  | 81 | dimers_comb_GM | 0.42% |
| 59 | dimers_comb_GN | 0.58% |  | 82 | dimers_comb_IT | 0.41% |
| 60 | dimers_comb_RS | 0.57% |  | 83 | dimers_comb_FP | 0.40% |
| 61 | dimers_comb_GG | 0.57% |  | 84 | dimers_comb_HK | 0.38% |
| 62 | dimers_comb_AC | 0.56% |  | 85 | dimers_comb_FM | 0.38% |
| 63 | dimers_comb_IL | 0.55% |  | 86 | dimers_comb_GT | 0.36% |
| 64 | dimers_comb_FL | 0.55% |  | 87 | dimers_comb_KR | 0.34% |
| 65 | dimers_comb_AM | 0.54% |  | 88 | dimers_comb_FH | 0.31% |
| 66 | dimers_comb_LL | 0.54% |  | 89 | dimers_comb_MM | 0.31% |
| 67 | dimers_comb_FI | 0.52% |  | 90 | dimers_comb_KM | 0.29% |
| 68 | dimers_comb_MW | 0.51% |  | 91 | dimers_comb_MY | 0.28% |
| 69 | dimers_comb_DR | 0.51% |  | 92 | dimers_comb_WW | 0.26% |
| 70 | dimers_comb_EF | 0.50% |  | 93 | dimers_comb_CC | 0.21% |
| 71 | dimers_comb_CY | 0.50% |  | 94 | dimers_comb_DW | 0.19% |
| 72 | dimers_comb_GH | 0.49% |  | 95 | dimers_comb_HW | 0.17% |
| 73 | dimers_comb_EP | 0.48% |  | 96 | tmhmm_pred_hel | 0.06% |

Table S8. Optimized hyperparameters of the Gradient Boosting classifier. In each stage, one or two parameters were optimized while the other parameters were left either at their final values from previous stages or at their default values if they had not been optimized previously. The parameters were first optimized using a large step size. Smaller steps were then used for refinement. The learning rate was lowered from the default value of 0.1 to 0.01 before optimizing the number of estimators. Parameters not mentioned here were left at their default values. The procedure is based on the *Complete Machine Learning Guide to Parameter Tuning in Gradient Boosting (GBM) in Python* by Aarshay Jain^[[1]](#footnote-1)^.

| **Stage** | **Parameter** | **Range** | **Step** | **Final value** |
| --- | --- | --- | --- | --- |
| 1 | n_estimators | 20-100 | 10 | -^a^ |
| 2 | max_depth | 3-17 | 2, 1 | 6 |
|  | min_samples_split | 100-1400 | 100, 50 | 1250 |
| 3 | min_samples_leaf | 1-160 | 10, 5 | 6 |
| 4 | max_features | 5-96 | 5 | 40 |
| 5 | subsample | 0.5-1 | 1/40 | 0.525 |
| 6 | learning_rate | -^b^ | -^b^ | 0.01 |
| 7 | n_estimators | 200-1800 | 200, 50 | 1500 |

^a^ The parameter was optimized again in the 7th stage, after which its final value was determined; ^b^ The learning rate was set to a fixed value; The final set of parameters was as follows: criterion='friedman_mse', init=None, learning_rate=0.01, loss='deviance', max_depth=6, max_features=40, max_leaf_nodes=None, min_impurity_decrease=0.0, min_impurity_split=None, min_samples_leaf=6, min_samples_split=1250, min_weight_fraction_leaf=0.0, n_estimators=1500, n_iter_no_change=None, presort='auto', random_state=9, subsample=0.525, tol=0.0001, validation_fraction=0.1, verbose=0, warm_start=False.

Table S9. Class disagreements between available training sets and the SoluProt test set when applying different binarization thresholds.

| **Dataset** | **FP1** | **FP2** | **FP3** | **FP4** | **FP5** | **FN1** | **FN2** | **FN3** | **FN4** | **FN5** | **E1** | **E2** | **E3** | **E4** | **E5** |
| --- | --- | --- | --- | --- | --- | --- | --- | --- | --- | --- | --- | --- | --- | --- | --- |
| PROSO II initial | 50 | 56 | 202 | 405 | 535 | 514 | 381 | 306 | 199 | 140 | 564 | 437 | 508 | 604 | 675 |
| DeepSol/ SKADE | 67 | 74 | 202 | 354 | 451 | 360 | 262 | 209 | 138 | 100 | 427 | 336 | 411 | 492 | 551 |
| SWI | 53 | 108 | 184 | 285 | 384 | 20 | 18 | 12 | 8 | 4 | 73 | 126 | 196 | 293 | 388 |
| SOLpro | 39 | 40 | 48 | 83 | 110 | 143 | 127 | 82 | 46 | 33 | 182 | 167 | 130 | 129 | 143 |
| SoluProt | 0 | 0 | 0 | 0 | 0 | 0 | 0 | 0 | 0 | 0 | 0 | 0 | 0 | 0 | 0 |

FP – false positives, FN – false negatives, E – total number of errors (FP + FN). The numerical suffix denotes the binarization threshold used for the SoluProt test set. For example, a binarization threshold of 2 means that all sequences with solubility scores of 2 or above are considered soluble, and all others are considered insoluble.

## References

Bjellqvist,B. *et al.* (1994) Reference points for comparisons of two-dimensional maps of proteins from different human cell types defined in a pH scale where isoelectric points correlate with polypeptide compositions. *Electrophoresis*, **15**, 529–539.

Bjellqvist,B. *et al.* (1993) The focusing positions of polypeptides in immobilized pH gradients can be predicted from their amino acid sequences. *Electrophoresis*, **14**, 1023–1031.

Cock,P.J.A. *et al.* (2009) Biopython: freely available Python tools for computational molecular biology and bioinformatics. *Bioinformatics*, **25**, 1422–1423.

Guruprasad,K. *et al.* (1990) Correlation between stability of a protein and its dipeptide composition: a novel approach for predicting in vivo stability of a protein from its primary sequence. *Protein Eng Des Sel*, **4**, 155–161.

Hirose,S. *et al.* (2011) Statistical analysis of features associated with protein expression/solubility in an in vivo Escherichia coli expression system and a wheat germ cell-free expression system. *J Biochem*, **150**, 73–81.

Kyte,J. and Doolittle,R.F. (1982) A simple method for displaying the hydropathic character of a protein. *J Mol Biol*, **157**, 105–132.

Smialowski,P. *et al.* (2012) PROSO II - a new method for protein solubility prediction. *FEBS J*, **279**, 2192–2200.

Vihinen,M. *et al.* (1994) Accuracy of protein flexibility predictions. *Prot Struct Funct Bioinf*, **19**, 141–149.

1. <https://www.analyticsvidhya.com/blog/2016/02/complete-guide-parameter-tuning-gradient-boosting-gbm-python/> [↑](#footnote-ref-1)
