# Supplementary figures and images for: SoluProt: prediction of soluble protein expression in Escherichia coli
Source: Bioinformatics. 2021 Jan 8;37(1):23–8. doi: 10.1093/bioinformatics/btaa1102 (PMC8034534; doi:10.1093/bioinformatics/btaa1102)

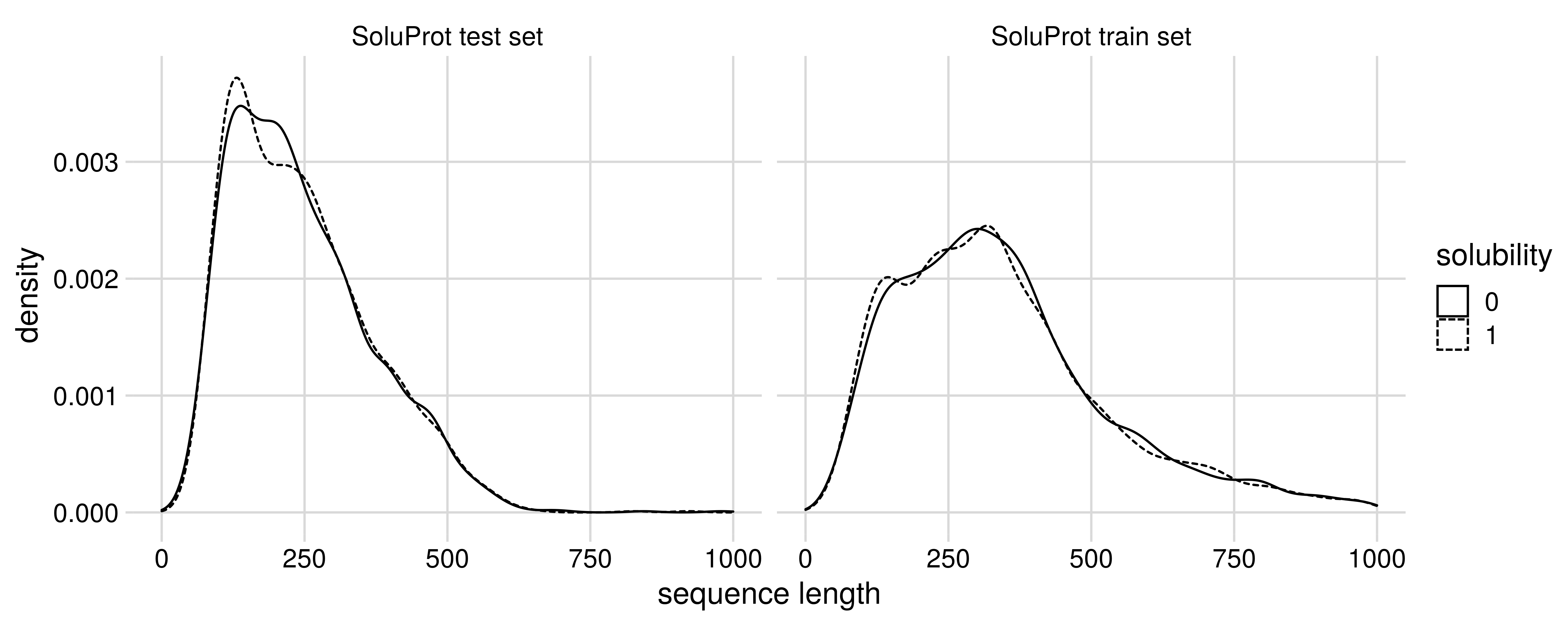

Supplement: btaa1102_Supplementary_Data [file btaa1102_supplementary_data.zip › Fig_S1.png]
